# Evaluation of Biocompatibility and Antagonistic Properties of Microorganisms Isolated from Natural Sources for Obtaining Biofertilizers Using Microalgae Hydrolysate

**DOI:** 10.3390/microorganisms9081667

**Published:** 2021-08-04

**Authors:** Olga Babich, Stanislav Sukhikh, Lyubov Dyshlyuk, Olga Shishko, Irina Milentyeva, Alexander Prosekov, Valery Pavsky, Svetlana Ivanova, Vyacheslav Dolganyuk

**Affiliations:** 1 Institute of Living Systems, Immanuel Kant Baltic Federal University, A. Nevskogo Street 14, 236016 Kaliningrad, Russia; olich.43@mail.ru (O.B.); stas-asp@mail.ru (S.S.); olesya.shishko@gmail.com (O.S.); dolganuk_vf@mail.ru (V.D.); 2 Department of Bionanotechnology, Kemerovo State University, Krasnaya Street 6, 650043 Kemerovo, Russia; soldatovals1984@mail.ru (L.D.); irazumnikova@mail.ru (I.M.); 3 Natural Nutraceutical Biotesting Laboratory, Kemerovo State University, Krasnaya Street 6, 650043 Kemerovo, Russia; 4 Laboratory of Biocatalysis, Kemerovo State University, Krasnaya Street 6, 650043 Kemerovo, Russia; a.prosekov@inbox.ru; 5 Department of General Mathematics and Informatics, Kemerovo State University, Krasnaya Street 6, 650043 Kemerovo, Russia; pavva46@mail.ru

**Keywords:** biofertilization, biocontrol, phytopathogen, rhizobacteria, plant growth promotion

## Abstract

Determination of the biocompatibility of microorganisms isolated from natural sources (Kemerovo Oblast—Kuzbass) resulted in the creation of three microbial consortia based on the isolated strains: consortium I (*Bacillus pumilus*, *Pediococcus damnosus*, and *Pediococcus pentosaceus*), consortium II (*Acetobacter aceti*, *Pseudomonas chlororaphis*, and *Streptomyces parvus*), and consortium III (*Amycolatopsis sacchari*, *Bacillus stearothermophilus*; *Streptomyces thermocarboxydus*; and *Streptomyces thermospinisporus*). The nutrient media composition for the cultivation of each of the three studied microbial consortia, providing the maximum increase in biomass, was selected: consortium I, nutrient medium 11; consortium II, nutrient medium 13; for consortium III, nutrient medium 16. Consortia I and II microorganisms were cultured at 5–25 °C, and consortium III at 50–70 °C. Six types of psychrophilic microorganisms (*P. pentosaceus*, *P. chlororaphis*, *P. damnosus*, *B. pumilus*, *A. aceti*, and *S. parvus*) and four types of thermophilic microorganisms (*B. stearothermophilus*, *S. thermocarboxydus*, *S. thermospinisporus*, and *A. sacchari*) were found to have high antagonistic activity against the tested pathogenic strains (*A. faecalis*, *B. cinerea*, *E. carotovora*, *P. aeruginosa*, *P. fluorescens*, *R. stolonifera*, *X. vesicatoria.* pv. *Vesicatoria*, and *E. aphidicola*). The introduction of microalgae hydrolyzate increased the concentration of microorganisms by 5.23 times in consortium I, by 4.66 times in consortium II, by 6.6 times in consortium III. These data confirmed the efficiency (feasibility) of introducing microalgae hydrolyzate into the biofertilizer composition.

## 1. Introduction

Agricultural production requires strategies to ensure the high efficiency of crop production without synthetic substances [1,2] and technologies for processing the crop to preserve it and increase the nutritional value and benefits of the resulting products [3,4,5,6,7,8].

The ability to tolerate biotic and abiotic stresses or nutrient-poor soils depends on the species of plants, and if their microbiome allows them to survive in harsh conditions [9]. This research aims to transfer these abilities to plants that lack thereof, including the biocontrol of potential pathogens [10,11]. By transferring one or more microbial species, more genetic material or gene numbers can be transferred compared to the transfer of a single gene, simultaneously improving several functions [12]. Compared to traditional artificial selection, in which a vast number of both specific and nonspecific genes are transferred to the recipient, genetic engineering of microorganisms transfers only a small block of the desired genes using both biolistic and Agrobacterium-mediated transformation. Changes in plant genomes occur either by genome targeting based on homologous recombination or nuclease-mediated site-specific genome modifications. It is possible to use recombinase-mediated site-specific genome integration and oligonucleotide-directed mutagenesis using microorganisms [13].

Recently, the term human microbiome and plant microbiome became entrenched in the minds of both researchers and consumers. The microbiome is a set of genomes of microorganisms in a specific habitat [14]. The plant microbiome consists of active organisms that change the physiology and development of plants, and increase plant resilience to pathogens, regulate the mechanisms of tolerance to various types of adverse effects, such as drought, salinity, or contaminated soils [9,10,15]. One or more microbial species perform these functions either in an additive or synergistic effect between two or more strains [16]. These types of bacteria can be used to create biofertilizers, by enhancing or activating the defense systems of other plant species that are susceptible to specific pathogens or abiotic factors, or to restore soil and increase yields [17,18,19].

The researchers of this study are interested in bacterial strains that survive in harsh conditions for their unique properties. The authors considered the biocatalytic potential of bacteria and their enzymes [20,21,22,23,24,25,26,27]. Thermophiles produce special proteins known as chaperonins that are heat stable and resistant to denaturation and proteolysis [28,29,30,31]. Psychrophiles possess adaptive mechanisms to perform their metabolic functions at low temperatures due to the unique abilities of their proteins and membranes [32,33,34].

Active bacteria application in agricultural technologies requires a deep understanding of the interaction mechanisms of these microorganisms and plants, both in vitro and in vivo [35,36,37]. Their industrial-scale production is part of bioprocess technology, which is quite well-developed, but this process must be adapted to the environmental conditions in each case. The search for microorganisms with unique properties that contribute to an increase in the yield of a wide range of crops is ongoing. However, the starting point is always to provide the necessary conditions for small-scale growth under controlled conditions. Stimulating biomass growth is one such condition, which is often included in the composition of the cultivation medium and has potential [9,38].

Algae are photosynthetic organisms, including eukaryotic green algae and gram-negative prokaryotic cyanobacteria, which have great potential as a bioresource for various industries. Microalgae have higher photosynthetic efficiency (carbon fixation) than terrestrial plants and produce a high biomass yield in a short time [39]. They are used in agriculture to provide nutrients and maintain soil fertility. Stimulating the activity of soil microorganisms by algae promotes plant growth and increases yields. Cyanobacteria provide nitrogen saturation through biological nitrogen fixation and enzymatic activity associated with converting and mobilizing various nitrogen forms. Both green algae and cyanobacteria are involved in the production of metabolites such as growth hormones, polysaccharides, antimicrobial compounds, etc., which play an essential role in the colonization of plants and the spread of microbes and eukaryotic communities in the soil. The use of algal biomass is an alternative to the efficient use of fertilizers [40].

Marine resources (microalgae) have a wide range of applications. Microalgae are the cheapest and best nitrogen source that does not pollute soil and water. Cyanobacteria are able to bind atmospheric nitrogen, which is useful for agriculture and has a positive effect on soil and plants, increasing fertility and yields. Algae-based biofertilizers are cost-effective and affordable, they are the best alternative to chemical nitrogen fertilizers [41].

This study aims to evaluate the biocompatibility and antagonistic properties of microorganisms isolated from natural sources of the Kemerovo Region to obtain biofertilizers with the addition of microalgae.

## 2. Materials and Methods

### 2.1. Sampling and Isolation of Microorganism Isolates

**Figure 1:**
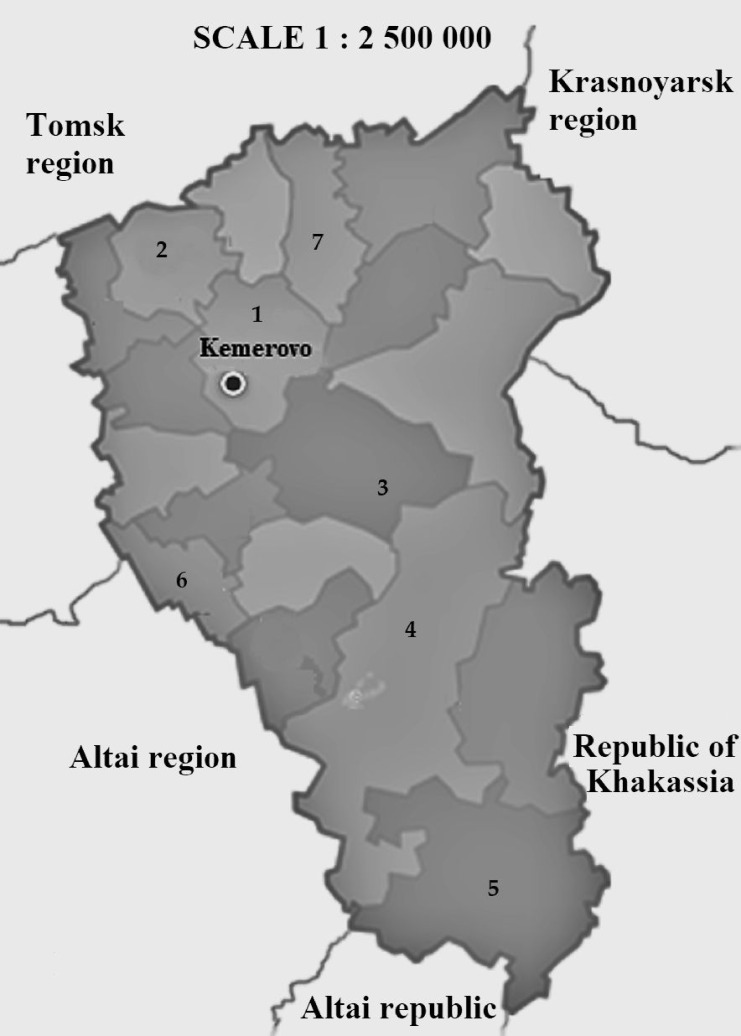
Places of microorganism sample collection: collection zone 1—Kemerovsky District, collection zone 2—Yashkinsky District, collection zone 3—Chebulinsky District, collection zone 4—Novokuznetsky District, collection zone 5—Tashtagolsky District, collection zone 6—Guryevsky District, collection zone 7—Izhmorsky District.

Soil, plant rhizosphere, agricultural waste, and compost were the materials for the isolation of pure cultures. Samples were collected in seven zones (collection zone 1—Kemerovsky District; collection zone 2—Yashkinsky District; collection zone 3—Chebulinsky District; collection zone 4—Novokuznetsky District; collection zone 5, Tashtagolsky District; collection zone 6, Guryevsky District; and collection zone 7—Izhmorsky District) of Kemerovo Region (Western Siberia, Russia) in April–May 2018 (Figure 1). Some samples were taken from the waste and composts of the plant growing enterprises of the Kemerovo Region: (A) LLC Niva (Gorskino, Guryevsky District, Russia), (B) ZAO Izhmorskaya prodovolstvennaya kompaniya (Izhmorsky District, Russia), (C) LLC Trud (Krasny Yar, Izhmorsky District, Russia), (D) JSC Sukhovsky (Kemerovo, Russia), (I) LLC KDV-Agro (Yashkinsky District, Russia).

The objects of the study were cultures of extremophilic microorganisms isolated from various natural sources of the Kemerovo Region (Table 1) and consortia composed of them. Strains of extremophilic microorganisms are indicated as *Acetobacter aceti* (EM1), *Amycolatopsis sacchari* (EM2), *Aspergillus flavus* (EM3), *Aspergillus niger* (EM4), *Bacillus caldotenax* (EM5), *Bacillus pumilus* (EM6), *Bacillus stearothermophilus* (EM7), *Enterococcus faecium* (EM8), *Halobacillus profundi* (EM9), *Micrococcus cryophilus* (EM10), *Pediococcus damnosus* (EM11), *Pediococcus pentosaceus* (EM12), *Pseudomonas chlororaphis* (EM13), *Pseudomonas syringae* (EM14), *Streptomyces parvus* (EM15), *Streptomyces thermocarboxydus* (EM16), *Streptomyces thermospinisporus* (EM17), and *Trichoderma lignorum* (EM18) were pre-selected for this study.

Microorganisms were isolated from natural sources by inoculation from the selected material into the thickness of the nutrient medium. The material was in a liquid state before inoculation. A measurement of 0.1, 0.5, or 1.0 mL of material was sampled from natural sources with a sterile graduated pipette and poured into sterile petri dishes. Then the material was poured into 15–20 mL of melted and cooled to 45–50 °C agar. Carefully shaking the cup, the material was mixed in it with circular movements on the table surface, reaching its uniform distribution in the medium. The dish was left closed until the agar had completely gelled and then turned upside down. Microorganisms were incubated at 20–25 °C for 24 h at pH = 5.3–5.4 to increase their biomass.

Microorganism isolates were obtained as follows: 0.5 M sodium hydroxide solution was added to the dry microorganism biomass and kept in a water bath for 10 min at 80 °C. The resulting extract was centrifuged for 20 min at a rotor speed of 3900 rpm. The supernatant was transferred to a clean chemical container. The obtained microorganism isolate was purified. A two-stage purification method was used: ultrafiltration and high-efficiency liquid chromatography (HPLC).

It was found that during ultrafiltration (membranes with a pore diameter of 100 kDa), a high efficiency of the purification process was achieved at an active acidity of 7.0 and a process pressure of 0.2 MPa. In this case, the concentration degree reached 80%.

Ultra-concentrated microorganism isolate samples were purified by HPLC according to the High-Performance Liquid Chromatography, General Pharmacopoeia (GPM.1.2.1.2.0005.15). This stage was performed on an LC-20 chromatograph (Shimadzu, Kyoto, Japan), eluting the isolate samples in a sodium chloride concentration gradient. Detection was carried out using a diode array detector in the detection range of 180 nm–900 nm, the flow rate of the eluent in all cases was 1 mL/min, elution was carried out in a gradient mode, the time and gradient were selected individually for each separation, the mixture of treated water (MQ purification level) and acetonitrile with the addition of 0.1% trifluoroacetic acid were used as solvents, separation was carried out on a reversed-phase Phenomenex column (Torrance, CA, USA) with dimensions of 250 × 2.5 mm, particle size 25 μm, sorbent silica gel modified C-18, with phenyl end-capping. The purified isolates of the microorganisms were used for further research.

### 2.2. Chemicals

The study used microalgae hydrolysate, purchased from Biorizon Biotech, Almería, Spain. All chemicals (analytical or better grade) used in this study were obtained from the Institute of Biotechnology of Kemerovo State University (Kemerovo, Russia).

### 2.3. Evaluation of Biocompatibility of Microorganisms

The biocompatibility was studied by co-cultivation on solid, dense nutrient media, typical for the studied strains. An overnight culture grown in a liquid medium and standardized according to the turbidity standard and was applied to the surface of a solid nutrient medium using a bacteriological loop with a 3 mm diameter. After applying a drop of the studied microorganism to a dense nutrient medium, a drop of another test culture was applied in the same volume but at a distance of 1–2 mm from the first one. After spreading, the second drop covered about half of the first drop. When superimposed, cultures developed in mutual presence (co-cultivation), competing with each other. After the second drop dried, the inoculated dishes were turned upside down and incubated at 37–39 °C in the air with a high carbon dioxide content. Each experiment was repeated, changing the position of the plates (to avoid the influence of the successive layering of drops on the nature of growth in the co-culture zone).

Superimposed drops of one culture, applied as described above, were used as control. The results were interpreted after 24 and 48 h of incubation. In the case of the growth suppression of a culture, the relationship between these cultures was considered antagonistic, and the cultures were classified as bioincompatible. Cultures are considered biocompatible in the case of the complete confluence of spots or increased growth of strains in the co-cultivation area (mutualism, synergism, satellitism). If one of the cultures in the co-cultivation zone emerges, inhibiting the growth of the second culture, regardless of the sequence of their introduction, this option is considered a weak antagonism. The presence of a clearly defined inhibition zone (growth inhibition) of one culture by other test cultures on the spot periphery was regarded as a sign of biological incompatibility.

### 2.4. Selection of Test Cultures and Assessment of Antagonistic Properties of Microorganisms

*Alcaligenes faecalis* B1820, *Botrytis cinerea* F1006, *Erwinia carotovora* B5194, *Pseudomonas aeruginosa* B6643, *Pseudomonas fluorescens* B954, *Rhizopus stolonifera* F520, *Xanthomonas vesicatoria* pv. *vesicatoria* B3740, *Erwinia aphidicola* B5034 are from the laboratory collections of the State Research Institute of Genetics and Selection of Industrial Microorganisms (Russia) and the Research Institute of Biotechnology of the Kemerovo State University (Russia) were used to evaluate the antagonistic activity of the isolated strains.

All strains were grown in a liquid nutrient media in 5 mL test tubes stationary for 3 days, then centrifuged, and the supernatant was filtered through 0.22 µm membrane filters. The resulting sterile solution of metabolites was used for experiments.

A suspension of overnight broth cultures grown on standard nutrient media was used. The number of microorganisms (titer) in the suspension was determined by optical density (OD) at a wavelength of 595 nm [42].

The antagonistic properties of the isolated microorganisms were evaluated in vitro by the diffusion method on a solid nutrient medium. The test strain was plated on an agar nutrient medium using a bacterial lawn technique, and simultaneously paper disks impregnated with the metabolites of the isolated microorganisms (10 μL/disk) were applied to the lawn. A disc with a nutrient medium was used as a control, and a disc with antibiotic ciprofloxacin (from a standard kit) was used as a reference drug. The plates were incubated at a temperature corresponding to the optimum growth temperature of each test strain for 24 h. The results were measured by the presence and size (in mm) of the transparent zone with an absence of microorganism growth around the disc.

### 2.5. Composition of Nutrient Media for Co-Cultivation of Microorganisms

A total of 18 culture media formulations were composed based on well-known culture media compositions to cultivate microorganisms (Table 2). All culture media components were weighed using a general-purpose laboratory balance with an accuracy of 0.01 g.

### 2.6. Statistical Analysis

Each experiment was repeated three times, and the data are expressed as means ± standard deviation. Data processing was carried out via the standard methods of mathematical statistics. The differences in the extracts were investigated by using a post hoc test (*p* < 0.05), and this test was performed in Statistica 10.0 (StatSoft Inc., 2007, Tulsa, OK, USA).

## 3. Results

Microorganism strains were isolated from the soil, rhizosphere, and the waste of plant growing enterprises of the Kemerovo Region (Table 1). Soil samples from the Kemerovsky and Novokuznetsky Districts are characterized by high salt content.

The results of studying the biocompatibility of the isolated strains are presented in Table 3.

The results of evaluating the antagonistic properties of the isolated microorganisms are presented in Table 4. Six types of psychrophilic microorganisms (*Pediococcus pentosaceus*, *Pseudomonas chlororaphis*, *Pediococcus damnosus*, *Bacillus pumilus*, *Acetobacter aceti*, and *Streptomyces parvus*) and four types of thermophilic microorganisms (*Bacillus stearothermophilus*, *Streptomyces thermocarboxydus*, *Streptomyces thermospinisporus*, and *Amycolatopsis sacchari*) were found to have high antagonistic activity against the tested pathogenic strains.

The pathogenicity of *Enterococcus faecium*, *Pseudomonas syringae*, *Aspergillus flavus*, and *Aspergillus niger*, despite their mild antagonistic and antibiotic properties, forced these strains to be excluded from further studies.

**Table 4:** Evaluation of the antagonistic and antibiotic properties of the isolated microorganisms.

Test Strains	Lysis Zone Diameter, mm																	
	EM1	EM2	EM3	EM4	EM5	EM6	EM7	EM8	EM9	EM10	EM11	EM12	EM13	EM14	EM15	EM16	EM17	EM18
1	19 *	21 *	0	0	2	20 *	21 *	11 *	0	7	24 *	20 *	17 *	5	15 *	22 *	19 *	0
2	22 *	21 *	0	0	3	21 *	17 *	10 *	0	0	23 *	21 *	16 *	4	18 *	24 *	18 *	0
3	25 *	22 *	6	0	0	21 *	26 *	0	7	0	15 *	22 *	21 *	0	19 *	23 *	23 *	5
4	22 *	19 *	4	8	0	19 *	19 *	0	0	0	19 *	17 *	25 *	0	24 *	20 *	20 *	6
5	15 *	23 *	18 *	5	30 *	0	9	15 *	4	24 *	5	0	0	23 *	15 *	17 *	21 *	25 *
6	18 *	20 *	19 *	0	16 *	8	3	18 *	2	15 *	0	7	13 *	20 *	6	21 *	24 *	18 *
7	21 *	20 *	12 * *	0	6	18 *	22 *	5	3	5	20 *	20 *	18 *	0	22 *	18 *	19 *	4
8	17 *	20 *	3	4	3	16 *	22 *	0	0	0	25 *	17 *	15 *	7	21 *	17 *	18 *	0
Control	0	0	0	0	0	0	0	0	0	0	0	0	0	0	0	0	0	0
Ciprofloxacin	25 *	27 *	26 *	24 *	25 *	21 *	22 *	24 *	20 *	21 *	26 *	25 *	22 *	23 *	28 *	22 *	25 *	24 *

**Figure 2:**
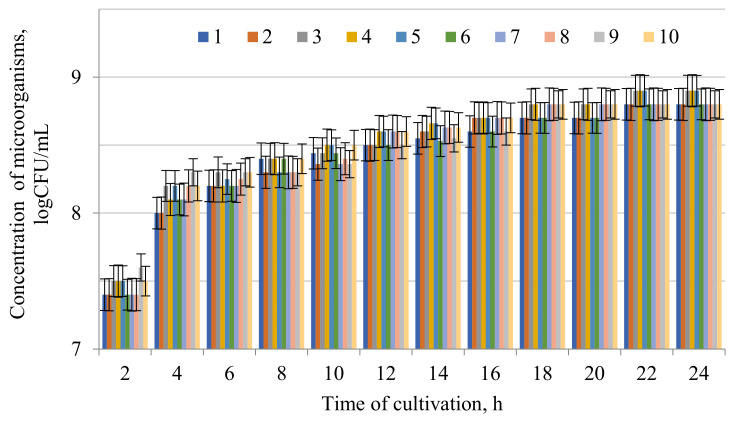
Growth of isolated microorganisms: 1—EM1, 2—EM2, 3—EM6; 4—EM7, 5—EM11, 6—EM12, 7—EM13, 8—EM15, 9—EM16, 10—EM7. Data presented as a mean ± SD (*n* = 3). The initial concentration was 6.0 × 10^3^.

EM1—*Acetobacter aceti*; EM2—*Amycolatopsis sacchari*; EM3—Aspergillus flavus; EM4—*Aspergillus niger*; EM5—*Bacillus caldotenax*; EM6—*Bacillus pumilus*; EM7—*Bacillus stearothermophilus*; EM8—*Enterococcus faecium*; EM9—*Halobacillus profundi*; EM10—*Micrococcus cryophilus*; EM11—*Pediococcus damnosus*; EM12—*Pediococcus pentosaceus*; EM13—*Pseudomonas chlororaphis*; EM14—*Pseudomonas syringae*; EM15—*Streptomyces parvus*; EM16—*Streptomyces thermocarboxydus*; EM17—*Streptomyces thermospinisporus*, EM18—*Trichoderma lignorum*; 1—*Alcaligenes faecalis*, 2—*Botrytis cinerea*, 3–*Erwinia carotovora*, 4—*Pseudomonas aeruginosa*, 5—*Pseudomonas fluorescens*, 6—*Rhizopus stolonifera*, 7—*Xanthomonas vesicatoria*. pv. *vesicatoria*, 8—*Erwinia aphidicola*. Values in columns followed by the symbol * differ significantly (*p* < 0.05) as assessed by the post hoc test (Tukey test). Data presented as a mean (*n* = 3). The results of the growth of isolated microorganisms, considering biocompatibility (Table 3) and antimicrobial activity (Table 4), are presented in Figure 2.

The following criteria were considered when creating the consortia:

- Specific properties possessed by the selected microorganisms;
- Peculiarities of metabolism of microorganisms isolated from natural sources of the Kemerovo Region;
- Conditions for cultivating microorganisms (cultivation time, temperature and pH value, nutrient medium composition).

Three consortia of microorganisms based on isolated strains were composed: consortium I (*Bacillus pumilus* (EM6), *Pediococcus damnosus* (EM11), *Pediococcus pentosaceus* (EM12)), consortium II (*Acetobacter aceti* (EM1), *Pseudomonas chlororaphis* (EM13), *Streptomyces parvus* (EM15)), and consortium III (*Amycolatopsis sacchari* (EM2), *Bacillus stearothermophilus* (EM7), *Streptomyces thermocarboxydus* (EM16), *Streptomyces thermospinisporus* (EM17)).

**Figure 3:**
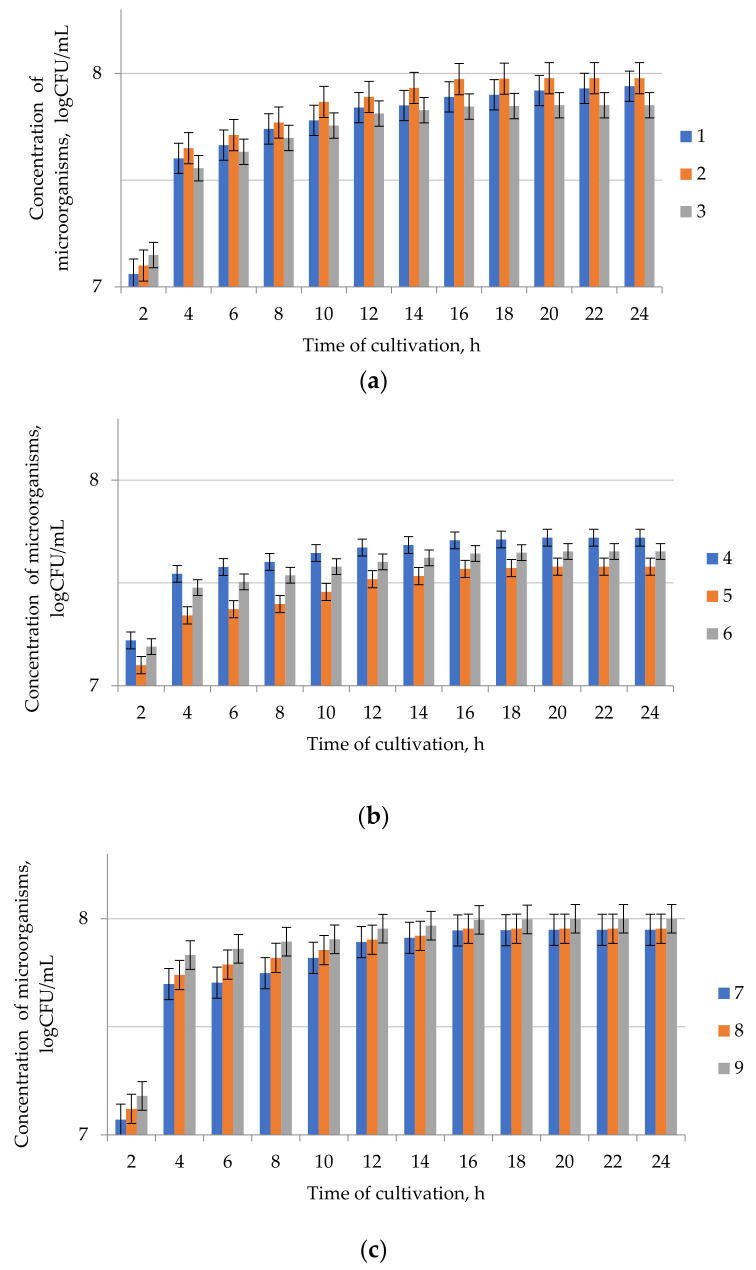
Dependence of the microorganism concentration in consortia (**a**) I, (**b**) II, and (**c**) III on the cultivation duration on different nutrient media: 1—media 1, 2—media 2, 3—media 3; 4—media 4, 5—media 5, 6—media 6, 7—media 7, 8—media 8, 9—media 9. The initial concentration was 5.8 × 10^3^, 6.0 × 10^3^, 5.7 × 10^3^ for consortia I, II, and III, accordingly. Data presented as a mean ± SD (*n* = 3).

**Figure 4:**
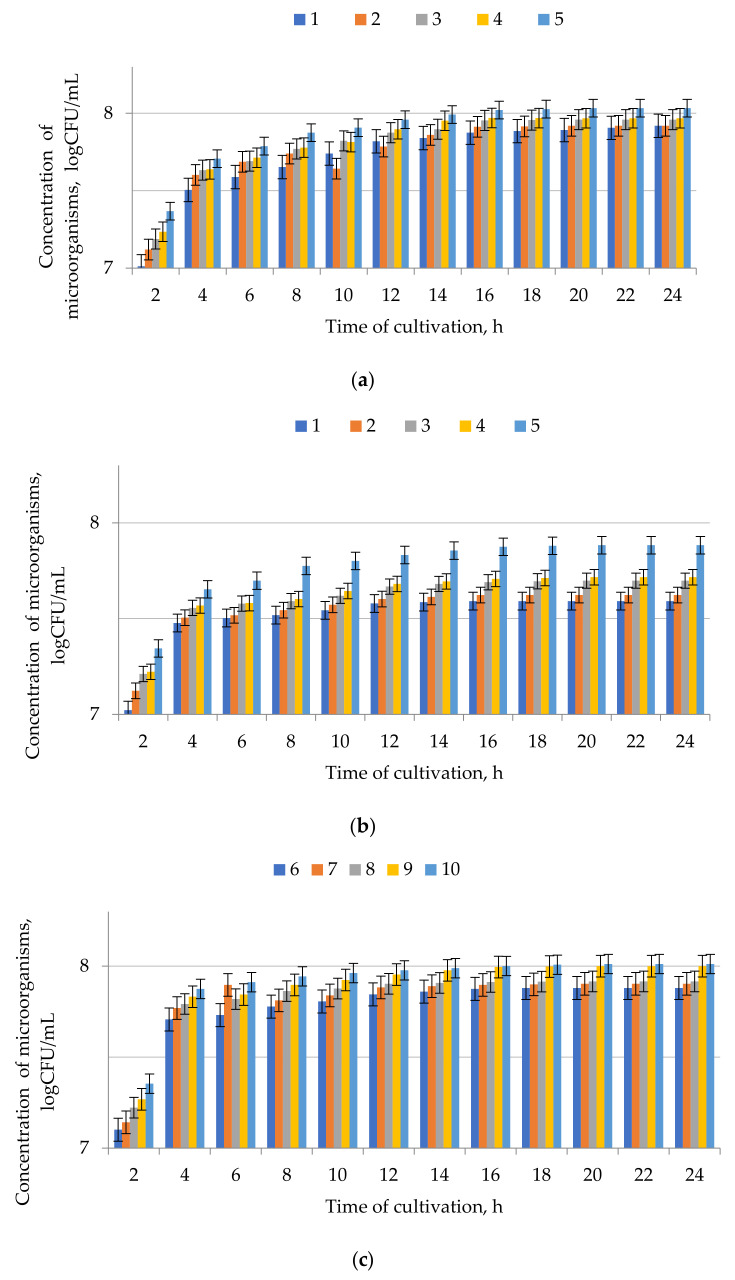
Dependence of the microorganism concentration in consortia (**a**) I, (**b**) II, and (**c**) III on the cultivation duration at different temperatures: 1—5, 2—10, 3—15, 4—20, 5—25, 6—50, 7—55, 8—60, 9—65, and 10—70 °C. The initial concentration was 5.8 × 10^3^, 6.0 × 10^3^, 5.7 × 10^3^ for consortia I, II, and III, accordingly. Data presented as a mean ± SD (*n* = 3).

**Figure 5:**
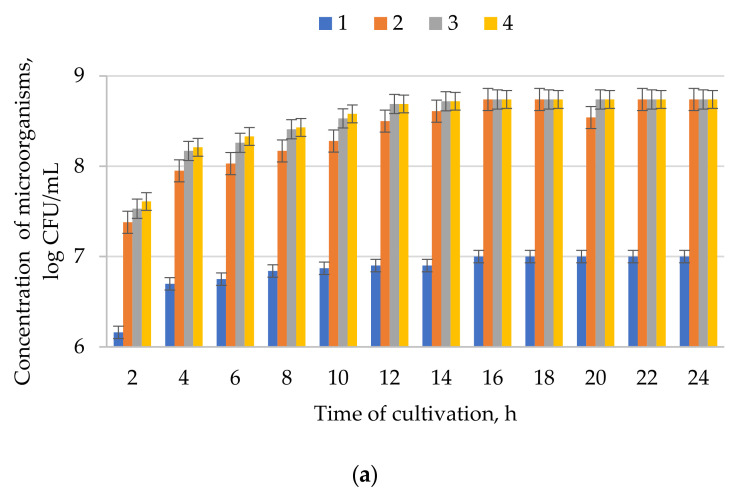

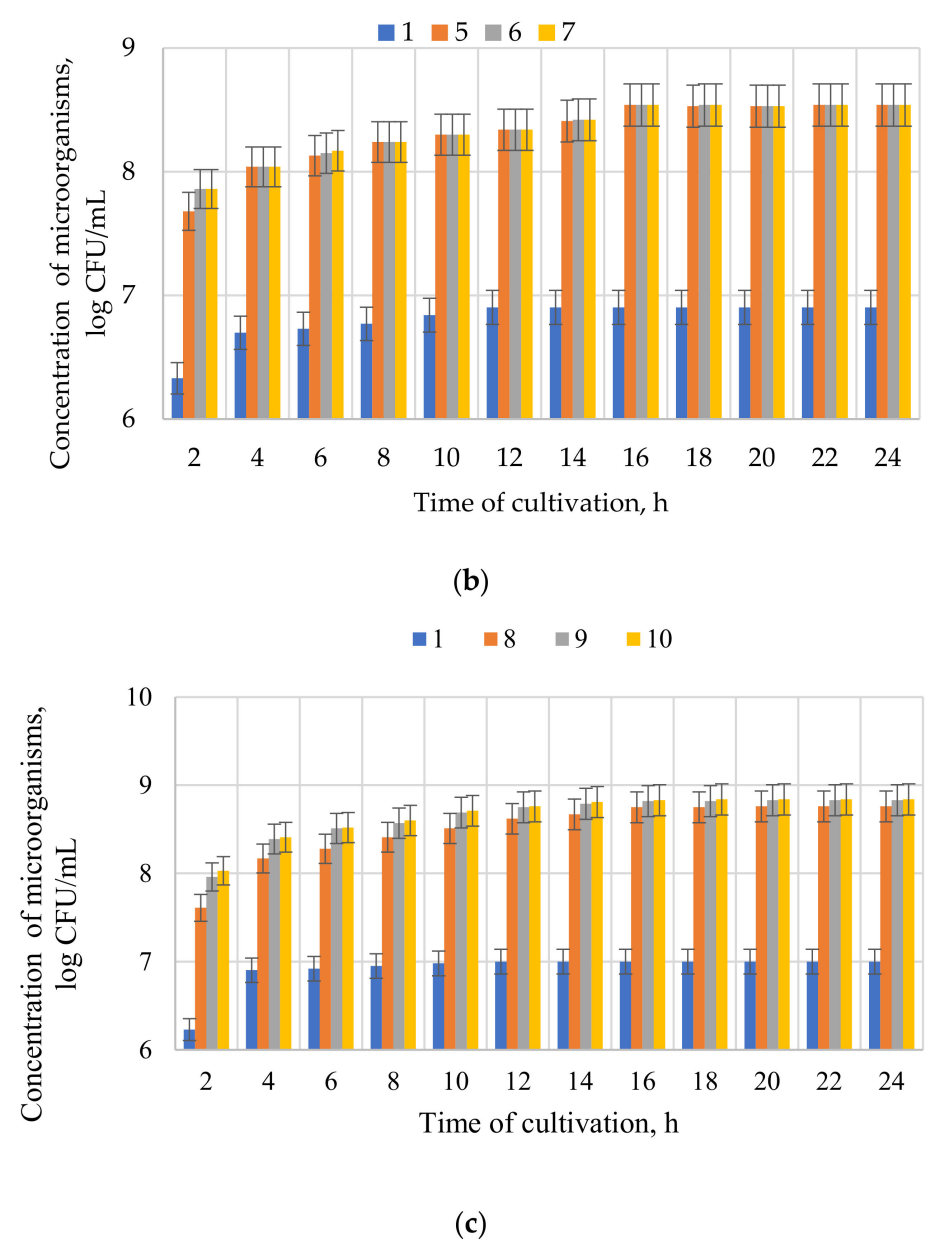
Dependence of the concentration of microorganisms in consortia (**a**) I, (**b**) II, and (**c**) III on the cultivation duration on different nutrient media with added microalgae: 1—control (no added microalgae hydrolysate), 2—medium 10, 3—medium 11, 4—medium12, 5—medium 13, 6—medium 14, 7—medium 15, 8—medium 6, 9—medium 17, 10—medium18. The initial concentration was 5.8 × 10^3^, 6.0 × 10^3^, 5.7 × 10^3^ for consortia I, II, and III, accordingly. Data presented as a mean ± SD (*n* = 3).

The results of determining the conditions for co-cultivation of the isolated microorganisms (nutrient medium composition, cultivation temperature, pH) are presented in Figure 3, Figure 4 and Figure 5. The optimal compositions of nutrient media (Table 2) for the cultivation of the three studied consortia of microorganisms were selected by varying the ratio of components and measuring the concentration of microorganisms. Consortium I was cultured for 24 h at 20 °C on nutrient media 1–3, consortium II for 24 h at 20 °C on nutrient media 4–6, and consortium III for 24 h at 55 °C on nutrient media 7–9.

Using the selected compositions of nutrient media, the effect of temperature on the process of co-cultivation of microorganisms, providing the maximum increase in biomass, was studied. Consortia I and II microorganisms were cultured at 5–25 °C, and consortium III at 50–70 °C. The results are presented in Figure 4.

The results of the study of the antagonistic activity of microbial consortia are presented in Table 5.

The results of evaluating the viability of consortia during their cultivation on nutrient media containing microalgae hydrolyzate are shown in Figure 4.

To increase the concentration of microorganisms that are part of the consortia and subsequently form the unique characteristics of biofertilizers, microalgae hydrolyzate was added to the nutrient media of the selected compositions as a nitrogen source (Table 2), and the concentration of microorganisms in the consortia was measured. The consortia were cultured at previously defined temperatures (10, 15, and 60 °C). The recommended nutrient medium for consortium I is nutrient medium11, for consortium II–nutrient medium 13, for consortium III–nutrient medium 17 containing 10–15 g/L of microalgae hydrolyzate. A further increase in the microalgae hydrolyzate content in the nutrient medium leads to only a slight increase in microorganisms.

## 4. Discussion

Microorganisms isolated from natural sources can survive in harsh environments at very high or low temperatures, pH, low humidity, or high salt concentrations (classified as thermophiles and psychrophiles, accordingly). These bacteria are molecularly adapted to withstand these harsh conditions [43].

When performing multiple tasks, consortia show better results than single microorganisms. The consortium composition must be selected in such a way that performance is optimized to achieve specific goals. In recent publications, metabolic modeling was successfully applied to develop a consortium [44,45,46]. The joint use of several strains of microorganisms enhances their unique qualities.

Maximum concentration (10.5 × 10^8^ CFU/mL, 7.5 × 10^8^ CFU/mL and 10.2 × 10^8^ CFU/mL) of microorganisms was achieved at 10, 15, and 60 °C, for consortia I, II, III, accordingly (Figure 3).

The study [47] examined the maximum specific growth rate of *Brochothrix thermosphacta*, the spoilage bacteria of cooked peeled shrimp, and *Lactococcus piscium* CNCM I-4031, a bioprotective strain, at various temperatures. It was found that *L. piscium* and *B. thermosphacta* were psychrotolerant, with T(min) = −4.8 and −3.4 °C, respectively, T(opt) = 23.4 and 27.0 °C, respectively, and T(max) = 27.2 and 30.8 °C, respectively. At an optimal temperature, the maximum concentration of microorganisms reaches values of 1 × 10^7–^1 × 10^8^ CFU/mL. The differences in the temperatures of the maximum concentration of microorganisms with the data obtained in our study are explained by the different types of studied microorganisms. The maximum concentration of microorganisms in our study was 10.5 × 10^8^ CFU/mL, 7.5 × 10^8^ CFU/mL, and 10.2 × 10^8^ CFU/mL.

The study [48] expanded the physiological study of *Bacillus* spp. SUBB01 with aeration at 100 rpm on various media, as well as at temperatures of 48, 50, 52, 53, and 54 °C. Bacterial growth was determined by counting viable and cultured cells (cells producing colony-forming units on Luria-Bertani plates and nutrient agar for 24 h). The maximum growth of microorganisms (1 × 10^7^ CFU/mL) was recorded at 54 °C. The obtained data correlate with our research. The maximum concentration of microorganisms in our study was 10.5 × 10^8^ CFU/mL, 7.5 × 10^8^ CFU/mL, and 10.2 × 10^8^ CFU/mL. Thus, we can conclude that we managed to obtain a substantially higher concentration of microorganisms.

The choice of phytopathogenic microorganisms (test cultures) is determined by the need to provide a reasonably wide range of biological control with the developed biofertilizers. *Pseudomonas fluorescens*, pathogenic bacteria causing soft rot on plant materials; *Botrytis cinerea*, the causative agent of brown rot of soft fruits and bulbous crops; *Rhizopus stolonifera*, the causative agent of rot in jackfruit, strawberry, sweet potato, peach and cotton; *Xanthomonas vesicatoria.* pv. *Vesicatoria*, the causative agent of diseases in sweet peppers and tomatoes; *Erwinia carotovora*, pathogen of most plants (carrots, potatoes, tomatoes, greens, squash, and other cucurbits, onions, green peppers, etc.); *Pseudomonas aeruginosa*, an opportunistic bacterium that causes nosocomial infections in humans; *Alcaligenes faecalis*, an opportunistic bacterium that causes intra-abdominal infections, septicemia, and meningitis in humans; *Erwinia aphidicola*, pathogenic bacteria causing soft rot on plant materials [6].

Microalgae and cyanobacteria have been identified as microorganisms with a high potential for biotechnological applications due to their remarkable ability to withstand harsh environmental conditions [48]. Being widespread in the surrounding world and photoautotrophic, they are widely used for the large-scale production of biologically active compounds and offer many advantages over other microorganisms. The introduction of microalgae hydrolyzate (Figure 3) increased the concentration of microorganisms by 2.23 times in consortium I, by 4.66 times in consortium II, by 3.6 times in consortium III. These data confirmed the efficiency (feasibility) of introducing microalgae hydrolyzate into the biofertilizer composition. The authors of [49] obtained similar results, but they managed to increase the growth of autotrophic microorganisms by only 1.5 times using the active biomass of microalgae obtained as a result of wastewater treatment. The inoculants *Lactobacillus bulgaricus*, *Issatchenkia orientalis*, and *Rhodopseudomonas palustris* in a ratio of 1:9 to microalgae of the Haihe River basin (Eastern Tianjin, China) were introduced into anaerobic sludge collected directly from treatment facilities (Tianjin, Binhijin) [8]. The content of organic matter in each of these samples was twice as high as recommended by the standard for biological organic fertilizers. The number of inoculated microorganisms (*Issatchenkia orientalis* and *Lactobacillus bulgaricus*) was higher than the recommended standard (0.2 × 10^9^ CFU/g); therefore, these agents are recommended as a microbial organic fertilizer.

## 5. Conclusions

The development of agriculture is associated with the search for new technologies aimed at increasing plant growth, improving soil fertility, and controlling the use of chemical fertilizers and pesticides. The necessary changes can be achieved by using the interaction of plants and microbes, which allows for the creation of environmentally friendly fertilizers with enormous potential.

The biocompatibility determination resulted in composing three microbial consortia based on strains isolated from natural sources of the Kemerovo Region: consortium I (*Bacillus pumilus*, *Pediococcus damnosus*, and *Pediococcus pentosaceus*), consortium II (*Acetobacter aceti*, *Pseudomonas chlororaphis*, and *Streptomyces parvus*), and consortium III (*Amycolatopsis sacchari*, *Bacillus stearothermophilus*, *Streptomyces thermocarboxydus*, and *Streptomyces thermospinisporus*).

The nutrient media composition providing the maximum increase in biomass of each of the three studied consortia was selected. Six types of psychrophilic microorganisms (*Pediococcus pentosaceus*, *Pseudomonas chlororaphis*, *Pediococcus damnosus*, *Bacillus pumilus*, *Acetobacter aceti*, and *Streptomyces parvus*) and four types of thermophilic microorganisms (*Bacillus stearothermophilus*, *Streptomyces thermocarboxydus*, *Streptomyces thermospinisporus*, and *Amycolatopsis sacchari*) were found to have high antagonistic activity against the tested pathogenic strains.

To increase the concentration of the consortia microorganisms and subsequently to form the unique characteristics of biofertilizers, microalgae hydrolyzate was added to the nutrient media of the selected compositions as a nitrogen source.

Microorganisms have great potential as biofertilizers. They can use CO_2_, water, and nutrients to convert solar energy into biomass. The effective use of microorganisms isolated from natural sources was documented in agricultural practice to reduce global warming by reducing carbon dioxide emissions. Some studies [50] show that the biomass of microorganisms can be used to improve food quality, physicochemical properties of soil, fight against diseases transmitted through the soil, add organic substances, release substances that promote growth, solubilize insoluble phosphates, and can be used as nutraceuticals and have pharmaceutical applications. Thus, biofertilizers prepared from microorganisms isolated from natural sources are cost-effective and environmentally friendly.

## Figures and Tables

**Table 1:** Microorganism strains isolated from natural sources (Kemerovo Region-Kuzbass).

Isolation Source	Collection Zone	Medium Temperature °C		
		0 °C	4 °C	10 °C
SL	1	-	EM12	EM3, EM12, EM18
SL	2	EM13	EM13	EM13
SL	3	-	EM15	EM15
SL	4	-	EM11	EM4, EM9, EM 11
SL	5	-	EM6	EM6
RP	1	-	EM8	EM8
RP	2	-	EM14	EM14
RP	3	-	EM1	EM1
RP	4	-	EM15	EM15
RP	5	-	EM1, EM6	EM1, EM6
PW	6 (A)	EM10	EM10, EM15	EM10, EM15
PW	7 (B)	-	EM10	EM10
PW	7 (C)	-	EM1	EM1
PW	1 (D)	-	EM1	EM1
PW	2 (E)	-	EM6	EM6
		40 °C	50 °C	60 °C
CP	6 (A)	EM16	EM16	EM16
CP	7 (B)	EM7	EM7	EM7
CP	7 (C)	EM5	EM5	EM5
CP	1 (D)	EM17	EM17	EM17
CP	2 (E)	EM2	EM2	EM2

SL—soil, RP–plant rhizosphere, PW–plant wastes, CP—compost. (A) LLC Niva (Gorskino, Guryevsky District), (B) ZAO Izhmorskaya prodovolstvennaya kompaniya (Izhmorsky District), (C) LLC Trud (Krasny Yar, Izhmorsky District), (D) JSC Sukhovsky (Kemerovo), (E) LLC KDV-Agro (Yashkinsky District). Microbial consortia were formed (in equal shares): I—EM6, EM11, EM12; II—EM1, EM13, EM15; III—EM2, EM7, EM16, EM17.

**Table 2:** Composition of nutrient medium for consortia cultivation.

Component g/L	No. of Nutrient Medium																	
	1	2	3	4	5	6	7	8	9	10	11	12	13	14	15	16	17	18
Casein hydrolyzate	20	15	10	-	-	-	-	-	-	15	15	15	-	-	-	-	-	-
Yeast extract	20	25	25	5	10	7	1	2.5	2	25	25	25	5	5	5	2	2	2
Gelatin	2.5	-	2	-	-	-	-	-	-	-	-	-	-	-	-	-	-	-
Glucose	5	3	5	-	-	-	-	-	-	3	3	3	-	-	-	-	-	-
Lactose	5	5	4	-	-	-	-	-	-	5	5	5	-	-	-	-	-	-
Sucrose	5	7	10	-	-	-	-	-	-	7	7	7	-	-	-	-	-	-
Sodium chloride	5	2	4	10	5	3	5	3	5	2	2	2	10	10	10	5	5	5
Sodium acetate	1.5	-	1	-	-	-	-	-	-	-	-	-	-	-	-	-	-	-
Ascorbic acid	0.5	-	-	-	-	-	-	-	-	-	-	-	-	-	-	-	-	-
Peptic digest of animal tissue	10	5	10	15	20	25	20	25	15	5	5	5	15	15	15	15	15	15
Beef infusion	500	300	300	-	-	-	-	-	-	300	300	300	-	-	-	-	-	-
Peotone	-	-	-	10	5	5	-	-	-	-	-	-	10	10	10	-	-	-
K2HPO4	-	-	2	1.5	2	2.5	1	2	1.5	-	-	-	1.5	1.5	1.5	1.5	1.5	1.5
(NH4)2HPO4	-	-	-	1	1.5	1.5	-	-	-	-	-	-	1	1	1	-	-	-
KH2PO4	-	-	-	0.5	1	1.5	-	-	-	-	-	-	0.5	0.5	0.5	-	-	-
MgSO4	-	-	-	0.5	0.5	1	-	-	-	-	-	-	0.5	0.5	0.5	-	-	-
Proteose peptone	-	-	-	5	10	7	-	-	-	-	-	-	5	5	5	-	-	-
Ammonium ferric citrate	-	-	-	0.5	1	1	0.5	1	1.5	-	-	-	0.5	0.5	0.5	1.5	1.5	1.5
Sodium thiosulfate	-	-	-	0.08	0.1	0.5	0.2	0.5	0.5	-	-	-	0.08	0.08	0.08	0.5	0.5	0.5
Glycerol	-	-	-	-	-	-	75	100	85	-	-	-	-	-	-	85	85	85
Corn extract	-	-	-	-	-	-	5	10	7.5	-	-	-	-	-	-	7.5	7.5	7.5
MgCl_2_	-	-	-	-	-	-	1.5	2	2	-	-	-	-	-	-	2	2	2
Agar-agar	15	25	20	15	20	20	25	20	20	25	25	25	15	15	15	20	20	20
Microalgae hydrolyzate	-	-	-	-	-	-	-	-	-	10	15	20	10	15	20	10	15	20
pH	6.5	7.2	7	7	6.8	7	7.5	7.2	7	-	-	-	-	-	-	-	-	-

**Table 3:** Biocompatibility of microorganisms.

Strains	EM1	EM2	EM6	EM7	EM11	EM12	EM13	EM15	EM16	EM17
EM1	WA	WA	BI	WA	BI	BI	BC	BC	WA	WA
EM2	WA	WA	WA	BC	WA	WA	WA	WA	BC	BC
EM6	BI	WA	WA	WA	BC	BC	BI	BI	WA	WA
EM7	WA	BC	WA	WA	WA	WA	WA	WA	BC	BC
EM11	BI	WA	BC	WA	WA	BC	BI	BI	WA	WA
EM12	WA	BI	BC	BC	BI	BI	WA	WA	WA	WA
EM13	BC	WA	BI	WA	BI	BI	WA	BC	WA	WA
EM15	BC	WA	BI	WA	BI	BI	BC	WA	WA	WA
EM16	WA	BC	WA	BC	WA	WA	WA	WA	WA	BC
EM17	WA	BC	WA	BC	WA	WA	WA	WA	BC	WA

BC—Biocompatible, BI—Bioincompatible, WA—Weak antagonism.

**Table 5:** Antagonistic activity of microbial consortia.

Test Strains	Lysis Zone Diameter, mm		
	ConsortiumI	ConsortiumII	ConsortiumIII
1	25	29	28
2	28	27	27
3	26	27	26
4	23	29	29
5	22	25	29
6	25	24	23
7	23	25	24
8	22	25	24
Control	0 *	0 *	0 *
Ciprofloxacin	24	28	25

1—*Alcaligenes faecalis*, 2—*Botrytis cinerea*, 3—*Erwinia carotovora*, 4—*Pseudomonas aeruginosa*, 5—*Pseudomonas fluorescens*, 6—*Rhizopus stolonifera*, 7—*Xanthomonas vesicatoria* pv. *vesicatoria*, 8—*Erwinia aphidicola*. Values in columns followed by the symbol * differ significantly (*p* < 0.05) as assessed by the post hoc test (Tukey test). Data presented as a mean (*n* = 3).

## Data Availability

Data are contained within the article.
